# Exercise Increases Adiponectin and Reduces Leptin Levels in Prediabetic and Diabetic Individuals: Systematic Review and Meta-Analysis of Randomized Controlled Trials

**DOI:** 10.3390/medsci6040097

**Published:** 2018-10-30

**Authors:** Tarik Becic, Christian Studenik, Georg Hoffmann

**Affiliations:** 1 Department of Pharmacology and Toxicology, Faculty of Life Sciences, University of Vienna, Althanstraße 14, 1090 Vienna, Austria; christian.studenik@univie.ac.at; 2 Department of Nutritional Sciences, Faculty of Life Sciences, University of Vienna, Althanstraβe 14, 1090 Vienna, Austria; georg.hoffmann@univie.ac.at

**Keywords:** exercise, adipokines, diabetes mellitus

## Abstract

It is speculated that lifestyle interventions known to improve diabetic metabolic state may exert their effects via adipokines. The aim of this systematic review and meta-analysis was to evaluate the chronic effects of physical exercise on adiponectin and leptin levels in adult prediabetic and diabetic individuals. PubMed, Embase, Scopus, The Cochrane Library, clinicaltrials.gov, and WHO Clinical Trials Registry were searched for randomized controlled trials. Pooled effects of interventions were assessed as mean difference (MD) with random effects model. Sensitivity analysis was conducted to test data robustness and subgroup analysis for study heterogeneity. Twenty-two trials with 2996 individuals were included in the meta-analysis. Physical exercise increased levels of adiponectin (MD: 0.42 µg/mL; 95% confidence interval (CI), 0.23, 0.60, *p* < 0.00001, *n* = 19 trials) and reduced leptin levels (MD: −1.89 ng/mL; 95% CI, −2.64, −1.14, *p* < 0.00001, *n* = 14 trials). These results were robust and remained significant after sensitivity analysis. Study heterogeneity was generally high. As for physical exercise modalities, aerobic exercise, but not other modalities, increased adiponectin and reduced leptin levels. In conclusion, physical exercise and, specifically, aerobic exercise, leads to higher adiponectin and lower leptin levels in prediabetic and diabetic adults. However, cautious interpretation of current findings is warranted.

## 1. Introduction

Diabetes mellitus represents a major global public burden. The number of diseased individuals has quadrupled between 1980 and 2014, rising from 108 million to 422 million [1]. Recent data demonstrate that about 150 million people worldwide suffer from diabetes, a number which is expected to double by 2025 [2]. Diabetic individuals are at a high risk of developing a range of complications, including heart disease, retinopathy, nephropathy, neuropathy, and diabetic foot complications [2]. An estimated 1.8 million deaths in 2012 were due to diabetes worldwide, whilst an additional 2.2 million deaths were associated with complications arising from higher-than-optimal blood glucose [1]. It is projected that, in 2030, diabetes mellitus will be the seventh leading cause of death globally [3]. Overweight and obesity and physical inactivity are major risk factors for developing the disease, as 9 out of 10 diabetic individuals in the United States are overweight or obese, and 4 out of 10 are physically inactive [4]. The pathological dysregulations that eventually lead to diabetes are preceded by prediabetes in most individuals [5]. This early phase represents a major opportunity for preventive interventions. The seminal Diabetes Prevention Program (DPP) showed that a lifestyle intervention including a physical activity component could significantly reduce the incidence of diabetes in high risk individuals, and the effects were even greater than with pharmacotherapy based on metformin [6]. In general, engaging in regular physical activity can reduce the risk of developing diabetes by 30–50% [7]. Even moderate intensity of physical activity, such as brisk walking, seems to offer protective benefits [8]. However, most individuals who are at risk or are already diagnosed with diabetes are not physically active on a regular basis [9]. Regular physical activity is also associated with improved glucose control in individuals already diagnosed with the disease. Physical exercise can reduce glycated haemoglobin HbA(1c) significantly, even in the absence of body weight changes [10], while higher intensity exercise is suggested to offer additional benefits on glycemic control and cardiorespiratory fitness [11]. Different training modalities are employed as strategies for managing abnormal glucose metabolism, including aerobic exercise, resistance exercise, and combined exercise. Both training modalities are important. For instance, aerobic exercise can prevent or change the course of peripheral diabetic neuropathy [12] and improve the cardiac autonomic nervous system function [13]. Resistance exercise was found to alter body composition in favor of lean muscle vs. adipose tissue which results in increased peripheral insulin sensitivity, among a plethora of other mechanisms [14]. In a randomized controlled trial, a combination of aerobic and resistance exercise was better in improving glycemic control than each modality alone [15]. Similar findings were found in a recent meta-analysis, where combined exercise was not only superior for glycemic control, but also in improving blood lipids in patients with diabetes [16]. However, another meta-analysis found that engaging in some form of physical activity is more important than choosing the type itself [17], which is important, given the rates of physically inactive diabetic and prediabetic individuals [18].

The physiological benefits of regular physical activity, as well as guidelines and recommendations on the type and amount, are provided in different guidelines for prevention and treatment of diabetes [18,19,20].

Adipose tissue is not considered an inert energy storage system any more. Since the discovery of leptin in 1994 [21], adipose tissue is regarded as a highly active endocrine system secreting a plethora of signaling molecules collectively known as adipokines [22]. Physical exercise has been found to affect adipose tissue, especially visceral adipose tissue, even in the absence of weight loss, and the effects are suggested to be even greater than with dietary restriction [23]. Leptin regulates a wide range of physiological mechanisms important for obesity and metabolic disorders, including energy balance, neuroendocrine function, and metabolic pathways. Leptin levels are primarily associated with the amount of fat tissue and energy balance. Leptin exerts is effects by binding to its receptor (ObR) in the hypothalamus, activating several signal transduction pathways, such as Janus kinase/signal transducer and activator of transcription-3 (JAK-STAT3), which is involved in the regulation of energy homeostasis, and phosphatidylinositol 3-kinase (PI3K), involved in the regulation of food intake and glucose homeostasis. Ultimately, leptin induces decreased food intake and increased energy expenditure, i.e., it has weight-reducing effects. However, in the state of leptin resistance, which is often found in type 2 diabetes, leptin cannot exert its effects, making these individuals resistant to the weight-reducing effects, even in the presence of hyperleptinemia [24]. Adiponectin is a hormone with anti-inflammatory and cardioprotective functions. Under normal conditions, it is secreted exclusively from adipose tissue. It is found abundantly in the plasma, accounting for 0.01% of plasma proteins in humans. Adiponectin exerts its effects by binding the receptors AdipoR1 and AdipoR2. The anti-inflammatory and cardioprotective properties are mostly due to inhibiting the expression of adhesion molecules, thereby reducing the adherence of monocytes to endothelial cells. In addition, adiponectin reduces plaque formation and increases plaque stability and nitric oxide (NO) production. In the liver, adiponectin reduces glucose output by inhibiting the expression of enzymes for gluconeogenesis. Its expression is paradoxically reduced in obesity, insulin resistance, and type 2 diabetes [25]. Both adipokines are clinically very relevant in prediabetes and diabetes, with the general perception that they exert contrary effects—leptin upregulates proinflammatory pathways which are associated with type 2 diabetes and cardiovascular disease, while adiponectin downregulates them [26]. Physical exercise has been found to affect adiponectin and leptin levels in a favorable manner [27,28], and the effect may be potentiated with dietary co-intervention [29].

The aim of this systematic review and meta-analysis was to synthesize data on the effects of physical exercise, including different exercise modalities, on adiponectin and leptin levels in prediabetic and diabetic individuals.

## 2. Materials and Methods

### 2.1. Literature Search

The following databases were searched until 1 March 2018 for randomized controlled trials: PubMed, Embase, Scopus, The Cochrane Library, clinicaltrials.gov, and WHO Clinical Trials Registry. Key words applied in the literature search were exercise, physical activity, training, adipokines, leptin, adiponectin, prediabetes, and diabetes. The reference sections of retrieved trials were also hand searched in order to identify further potentially relevant trials. Systematic reviews and meta-analysis which were identified were also hand-searched for additional trials. The search strategy for PubMed is provided in the Supplementary Material (Figure SX). Our systematic review is registered in PROSPERO (CRD42018098633).

### 2.2. Study Selection

Studies were included if they were (i) randomized controlled trials involving adults (minimum 18 years of age) with prediabetes (insulin resistance, impaired glucose tolerance, impaired fasting glucose) or type 2 diabetes; (ii) used physical exercise in supervised form, including different exercise modalities (aerobic, resistance, and concurrent exercise) or provided exercise advice to enrolled individuals; (iii) an intervention time of minimum 4 weeks; iv) evaluated adiponectin and/or leptin as outcomes; and (v) reported change in means or baseline and postintervention means with standard deviations for the intervention and control group, or values from which these could be calculated.

Studies were excluded if they (i) lacked a control group; (ii) included individuals with type 1 diabetes; (iii) involved a confounding co-intervention other than diet, e.g., a drug cotreatment; (iii) lacked sufficient information on the outcomes of interest; (iv) were conference abstracts, reviews, case reports, commentaries; and (v) were duplicate publications.

### 2.3. Risk of Bias Assessment

The Cochrane risk of bias tool [30] was used to evaluate the risk of bias of included trials (low, unclear, high) for the following study characteristics: random sequence generation (selection bias), allocation concealment (selection bias), blinding of participants and personnel (performance bias), blinding of outcome assessment (detection bias), incomplete outcome data (attrition bias), selective reporting (reporting bias), and other bias.

### 2.4. Data Extraction and Analysis

The following data, that were abstracted from every trial, included first author’s last name, publication year, modality of physical exercise, sample size, sex distribution, baseline characteristics (age, body mass index (BMI)), medical condition, number of training sessions per week, intervention duration, dietary co-intervention, and outcome parameters. If several time points were reported for an outcome, then data from the longest follow-up time period was taken. If the trials included multiple different physical exercise interventions, then data were extracted from every intervention arm.

Where reported, changes in group means and corresponding standard deviations (SDs) for levels of adiponectin and leptin were extracted for the intervention and control groups. Otherwise, changes were calculated as the difference between the post-intervention and baseline mean; in this case, SD was calculated for each group, assuming that r = 0.5 [31], as:

√[(SDbaseline)2 + (SDend of treatment)2 − (2r × SDbaseline × SDend of treatment)]

If medians or interquartile ranges were reported instead of means, then mean values and SDs were calculated as proposed by Hozo et al. [32].

The statistical analysis was done with Review Manager 5.3 of the Cochrane Collaboration Group [33]. The analysis was done by using the inverse-variance random effects model [34]. Effect size of the intervention was calculated as the pooled estimates of the weighted mean differences (WMD) between the intervention and control groups.

Study heterogeneity was measured by Higgins I^2^ statistic [35], where a value higher than 50% was considered to represent considerable heterogeneity.

Subgroup analyses were conducted according to preset criteria: (i) intervention duration, where we applied 12 weeks as cut-off [36]; (ii) dietary co-intervention, as it can modulate the effect of exercise [29]; and (iii) number of training sessions per week, based on recent recommendations of the American Diabetes Association [37].

Sensitivity analysis through the leave-one-out method was employed to verify the robustness of data by removing one trial at a time from the meta-analysis, and recalculating the effects with the remaining trials. In addition, sensitivity analysis was also done by leaving out studies which involved prediabetics, and by excluding trials with two or more defined areas of high risk of bias.

Publication bias was inspected through the funnel plot method, where the differences in mean changes are plotted against their standard errors, in order to determine the precision of the studies.

## 3. Results

### 3.1. Characteristics of Included Trials

Once all selection criteria were applied, a total of 22 studies with 2996 individuals were included in the analysis [38,39,40,41,42,43,44,45,46,47,48,49,50,51,52,53,54,55,56,57,58,59]. Figure S1 provides an overview of the search strategy.

A total of 19 studies reported adiponectin levels as outcome, while 14 studies reported leptin levels (Table 1). Most studies employed a structured physical exercise program, the majority of which was aerobic exercise. Three studies [39,58,59] provided exercise advice. Multiple different exercise modalities were used in three studies [39,40,51]; therefore, multiple effect sizes were extracted from these studies. Most trials included both sexes; whilst five [38,41,42,45,53] enrolled men only and one [51] women only. The participants were heterogeneous in terms of age (overall range: 36–66 years). Mean BMI of all groups was higher than 25 kg/m^2^ or 30 kg/m^2^, hence, the participants included were overweight and obese. The absolute majority of trials enrolled individuals with type 2 diabetes, while three [52,53,59] included prediabetic individuals. The number of training sessions per week was in the range of 2–6. The length of intervention duration, in weeks, ranged between six and 104. Five out of 22 studies [44,48,54,58,59] provided dietary treatment as part of the intervention. Figure S2 provides an overview of the risk of bias.

### 3.2. Influence of Exercise on Adiponectin and Leptin Levels

**Figure 1:**
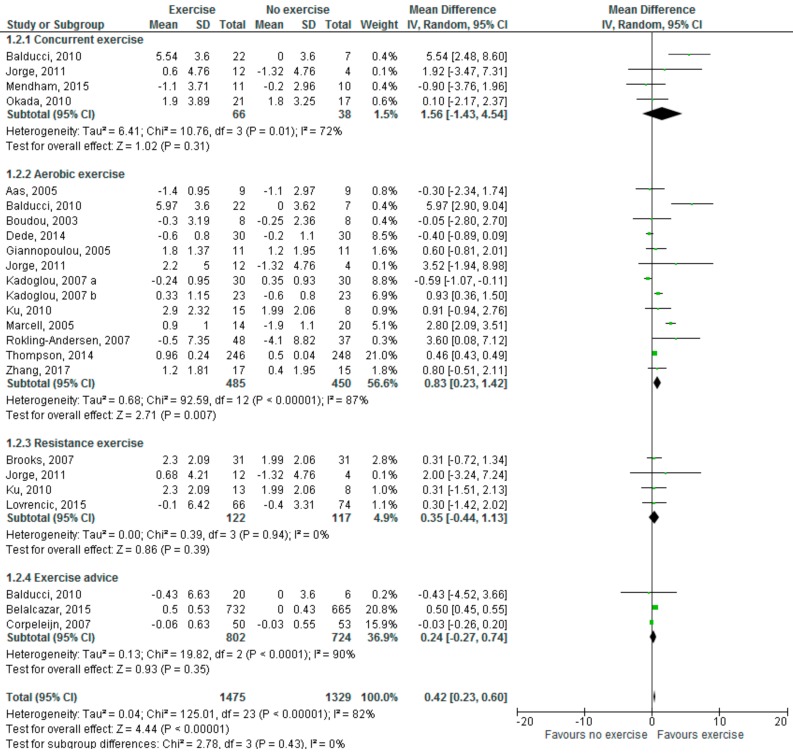
Effects of physical exercise, including different modalities, on adiponectin levels (µg/mL). Forest plot shows pooled mean differences with 95% confidence intervals (CI) for 24 effect sizes pooled from 19 trials (two separate effect sizes were pooled for different exercise modalities from Jorge [40] and Ku [51], and three from Balducci [39]). The green colored square represents the point estimate of the effect of the intervention for each trial. The horizontal line joins the upper and lower limits of the 95% CI of the effects. The square area represents the relative weight of the trial in the meta-analysis. The black colored diamond at the bottom represents the pooled mean difference with 95% CI for all study groups.

The present meta-analysis shows that physical exercise significantly increases adiponectin levels (Figure 1) in prediabetic and diabetic individuals (mean difference (MD): 0.42 µg/mL; 95% CI 0.23, 0.60, *p* < 0.00001), but significant study heterogeneity was found (I^2^ = 82%).

**Figure 2:**
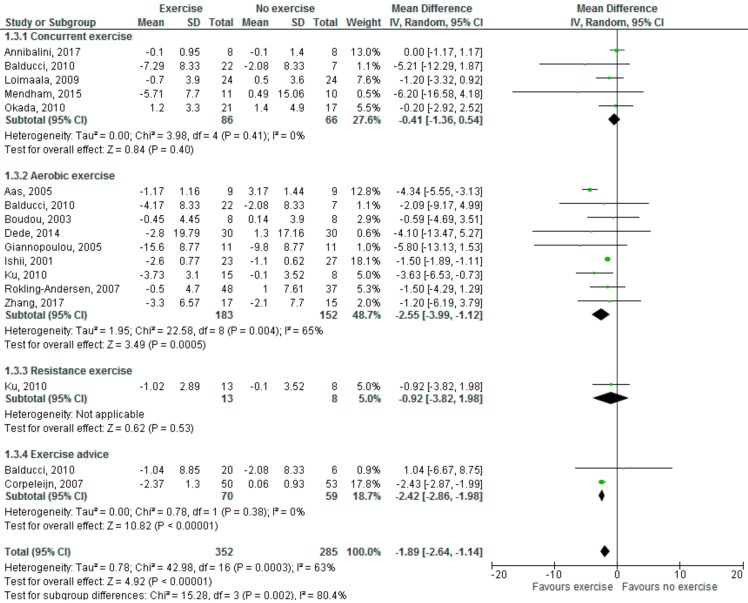
Effects of physical exercise, including different modalities, on leptin levels (ng/mL). Forest plot shows pooled mean differences with 95% confidence intervals (CI) for 17 pooled effect sizes from 14 trials (two separate effect sizes were pooled for different exercise modalities from Ku [51] and three from Balducci [39]). The green colored square represents the point estimate of the effect of the intervention for each trial. The horizontal line joins the upper and lower limits of the 95% CI of the effects. The square area represents the relative weight of the trial in the meta-analysis. The black colored diamond at the bottom represents the pooled mean difference with 95% CI for all study groups.

As shown in Figure 2, physical exercise significantly reduced leptin levels (MD: −1.89 ng/mL; 95% CI, −2.64, −1.14, *p* < 0.00001); here, as well, high heterogeneity was found (I^2^ = 63%).

With regards to effects of different exercise modalities, aerobic exercise significantly increased adiponectin levels (MD: 0.83 µg/mL; 95% CI, 0.23, 1.42, *p* = 0.007, I^2^ = 89%), but neither concurrent/resistance exercise nor exercise advice significantly affected adiponectin levels. With regards to leptin levels, both aerobic exercise (MD: −2.55; 95% CI, −3.99, −1.12, *p* = 0.0005, I^2^ = 65%) and exercise advice (MD: −2.42; 95% CI, −2.86, −1.98, *p* < 0.00001, I^2^ = 0%) led to a significant reduction in serum levels.

### 3.3. Sensitivity Analysis

As described above, each study was removed once from the meta-analysis, and the effects recalculated with the remaining studies. No major changes in the effect size were found, suggesting a robustness of data in the primary analysis. For adiponectin, the minimal effect size was found once Marcell [52] was removed from the meta-analysis (MD: 0.30; 95% CI, 0.14, 0.46, *p* = 0.0003, I^2^ = 74%), while removing Thompson [54] from the analysis generated the largest effect size (MD: 0.64; 95% CI, 0.24, 1.04, *p* = 0.002, I^2^ = 82%).

Once Aas [44] and Kadoglu [50] were removed from the analysis, there was an increase in effect size, but the effects remained statistically significant (MD: 0.65 µg/mL; 95% CI, 0.21, 1.09, *p* = 0.004, I^2^ = 84%).

Removing studies which involved prediabetic subjects [52,53,59] from the analysis did not change the overall effect size of physical exercise on adiponectin levels (MD: 0.37 µg/mL; 95% CI, 0.21, 0.53, *p* < 0.00001, I^2^ = 68%). However, removing Corpeleijn [59] from the analysis doubled the effect size of exercise advice, reduced study heterogeneity to 0%, and the effect was statistically significant (MD: 0.50 µg/mL; 95% CI, 0.45, 0.55, *p* < 0.00001, I^2^ = 0%). For leptin, removing Aas [44] decreased the effect size the most, but also reduced heterogeneity below 50% (MD: −1.55; 95% CI, −2.20, −0.91, *p* < 0.00001, I^2^ = 44%). Once Annibalini [38] was left out from the analysis, the largest effect size was seen for leptin (MD: −2.19; 95% CI, −2.92, −1.46, *p* < 0.00001, I^2^ = 53%). Removing the two studies with high risk of bias [44,48] from the analysis reduced study heterogeneity both for the overall effect of physical exercise (MD: −1.54; 95% CI, −2.45, −0.63, *p* = 0.0009, I^2^ = 38%), and in the aerobic exercise subgroup (MD: −2.28; 95% CI, −3.86, −0.69, *p* = 0.005, I^2^ = 0%). Limiting the analysis to type 2 diabetics only did not change the overall effects of physical exercise (MD: −1.81; 95% CI, −2.84, −0.79, *p* = 0.0005, I^2^ = 59%), but the results in the exercise advice subgroup became non-significant after removing Corpeleijn [59] (MD: −1.04; 95% CI, −6.67, 8.75, *p* = 0.79).

### 3.4. Subgroup Analysis

Subgroup analyses were conducted for intervention duration, dietary co-intervention, and the number of training sessions per week (Table 2). For the number of training sessions per week, Okada [43] and Giannopoulou [47] were excluded from the analysis as they did not specify the exact number of training sessions per week; all exercise advice studies were excluded, as well.

A statistically significant increase in adiponectin levels was found across all subgroups, but study heterogeneity remained high. Interestingly, for intervention duration, studies which lasted ≥12 weeks produced an approximately 5-fold higher increase in adiponectin levels than studies with longer duration (MD: 0.12 vs. 0.49 µg/mL). Interventions that did not include a dietary co-intervention produced a double higher increase in adiponectin levels (MD: 0.99 vs. 0.40 µg/mL). Limiting the number of training sessions to three times per week or less led to approximately 2-fold higher adiponectin levels increase than a higher number of training sessions (MD: 1.70 vs. 0.76). However, statistically significant differences were found only between the subgroups for intervention duration (*p* = 0.0009).

As for leptin levels, a significant reduction was found across all subgroups as well. Intervention duration >12 weeks led to a higher reduction than shorter duration (MD: −2.69 vs. −1.50 ng/mL). Dietary co-intervention tripled the reduction effect on leptin levels (MD: −2.60 vs. −0.87 ng/mL). Exercising three or less times per week reduced leptin more than a higher frequency (MD: −2.27 vs. −1.72 ng/mL). Interestingly, in subgroups with ≤12 weeks treatment duration, no dietary co-intervention, and >3 training sessions per week, there was very low study heterogeneity. Based on the test for subgroup differences, statistically significant differences were found for the intervention duration and dietary co-intervention subgroup analyses.

### 3.5. Publication Bias

**Figure 3:**
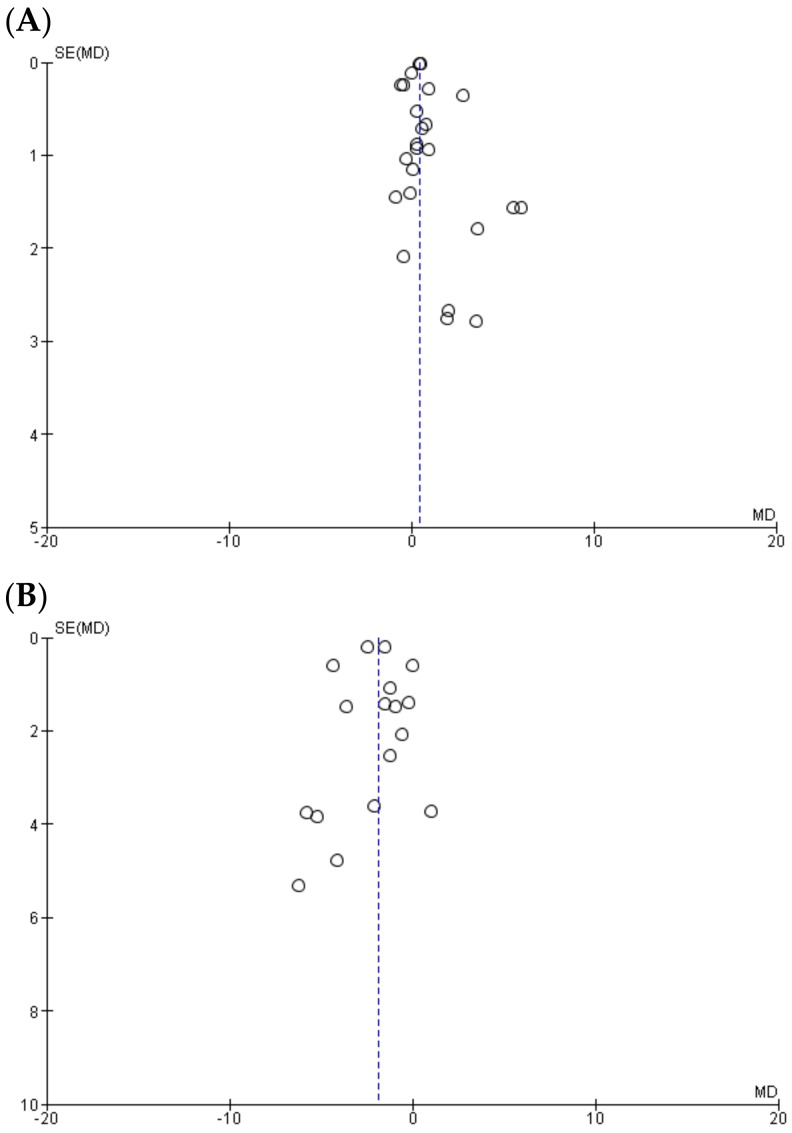
Funnel plot showing study precision against the mean difference effect estimate with 95% confidence interval for (**A**) adiponectin and (**B**) leptin. SE—standard error, MD—mean difference.

Visually inspecting the funnel plots both for adiponectin (Figure 3A) and leptin (Figure 3B) revealed a moderate asymmetry, such that it cannot be excluded that a publication bias, such as not publishing indecisive data, could have affected the results of the present meta-analysis.

## 4. Discussion

Our meta-analysis shows that physical exercise and, specifically, aerobic exercise, increased adiponectin and reduced leptin levels in prediabetic and diabetic individuals.

As global diabetes rates continue to increase and the underlying conditions, such as obesity and prediabetes, are on the rise, it is of utmost importance to identify strategies for their successful management. Although numerous pharmacotherapy options are available for type 2 diabetes, lifestyle interventions always form an integral part of a diabetes management plan. A preponderance of evidence demonstrates the benefits of physical exercise for a whole set of criteria relevant for prediabetes and diabetes, including better immediate glucose clearance from the blood and long-term improvements of blood sugar and HbA(1c) levels, favorable body composition changes, increased aerobic capacity, better cardiovascular outcomes, and overall reduced morbidity and mortality [1,7,10,11,16].

Adipokines might represent a possible explanation when it comes to the mechanisms mediating the beneficial effects of physical exercise on impaired glucose metabolism.

Hypoadiponectinemia has been associated with impaired glucose regulation, inflammation, obesity, atherosclerosis, and type 2 diabetes [60]. Increasing adiponectin levels has been associated with a lower risk for developing diabetes across populations in a dose–response relationship [61]. In diabetic individuals, enhancing adiponectin levels has emerged as a promising strategy due to its beneficial clinical effects, including anti-inflammatory [62], insulin-mimicking, and insulin-sensitizing [62] properties. Physical exercise exerts increasing effects on adiponectin comparable to the one of some anti-diabetic drugs [63]. Our meta-analysis shows that physical exercise, in general, and aerobic exercise, significantly increase adiponectin levels in prediabetic and diabetic adults. These results are in line with previous meta-analysis done in overweight and obese individuals [64] and, also, with systematic reviews [36]. Another meta-analysis from 2014 [65] did not find significant changes in adiponectin levels in response to physical exercise in diabetic individuals; however, this meta-analysis included much less studies than ours, it did not include prediabetic individuals and, also, considered interventions which included drug co-treatment. The robustness of the data was demonstrated in the sensitivity analysis, where it was demonstrated that the results were not dependent on any single study included. Interestingly, exercise advice also led to a significant increase in adiponectin levels once the analysis was constrained to diabetic individuals only. However, high inter-study heterogeneity was generally found. In the subgroup analysis, intervention duration was the only characteristic with significant differences between the subgroups and might, therefore, explain, in part, the heterogeneity. Notably, high study heterogeneity for adiponectin levels was found in previous meta-analysis as well [65].

Leptin is an “adipostat” regulating body fat mass, whose concentration changes with changing fat stores under physiological conditions, with the ultimate goal of maintaining stable body energy stores [66]. However, in type 2 diabetes, leptin levels are generally higher independently of body fat mass [67]. This hyperleptinemia is regarded as a marker of leptin resistance, a condition where tissues do not respond normally to leptin [68]. Leptin resistance in diabetes further aggravates the disarrangements in glucose metabolism [69] and is a significant factor in the development of diastolic function and heart failure [70]. Reducing leptin levels, inflammation, and oxidative stress, are suggested to improve overall leptin sensitivity [71]. Physical exercise is known to reduce oxidative stress and inflammation [72]. In our meta-analysis, we show that physical exercise, especially aerobic exercise, significantly reduces leptin levels. The data were robust, as no significant changes of effect size were found in the sensitivity analysis. However, after leaving out studies with a high risk of bias, study heterogeneity was reduced to below 50%. Exercise advice also led to a significant reduction in leptin levels, but the effect disappeared once the analysis was constrained to only type 2 diabetic individuals, which might imply that exercise advice is able to reduce leptin levels primarily in prediabetic individuals. In general, our results are in accordance with previous work, which also reported significant leptin reduction following physical exercise interventions [64,65]. The subgroup analysis showed that intervention duration and presence of dietary co-intervention are variables with statistically significant differences between subgroups. Leptin levels are highly sensitive to energy balance, such that negative energy balance through caloric deficit leads to a reduction in circulating leptin [73]. It is, therefore, plausible that the addition of dietary co-intervention potentiates the reducing effects of exercise on leptin through creating a larger negative energy balance, and the effects are greater with the duration of the negative energy state.

Interestingly, the present meta-analysis revealed that aerobic exercise, but not other exercise modalities, lead to significant increase in adiponectin levels and a reduction in leptin levels. This was also found in previous meta-analyses [64,65], and might be explained through greater negative energy balance induced by aerobic exercise as compared to other exercise modalities [74], but also an overall greater effect of aerobic exercise on body weight and fat mass [75].

However, the present study has several limitations. The risk of bias could not be assessed across many of the preset criteria. In addition, high study heterogeneity was found, and could not be fully explained in the subgroup analyses. Furthermore, the population set analyzed was heterogeneous in terms of age distribution, BMI, and clinical condition. Also, the design of physical exercise interventions differed, e.g., in terms of session duration and intensity. For aerobic exercise, we could not make a differentiation between potentially different effects of interval vs. continuous exercise. Some studies also had very small study groups, which tends to produce more extreme effects. Publication bias could also not be excluded.

In conclusion, the present systematic review and meta-analysis shows that exercise represents a viable strategy to increase adiponectin and reduce leptin levels in prediabetic and diabetic individuals. However, a cautious interpretation is warranted.

## Figures and Tables

**Table 1:** Study characteristics. Values expressed are mean ± standard deviation (SD), unless indicated otherwise.

Author	Sample Size; % Female	Age (Years, Mean ± SD)	BMI (kg/m2, Mean ± SD)	Medical Condition	Training Sessions/Week	Intervention Duration in Weeks	Training Characteristics	Dietary Co-Intervention	Change in Outcomes as Compared to Baseline (Mean ± SD) ^†^
**Concurrent Exercise**									
Annibalini, 2017 [38]	16; 0%	I: 57 ± 9.1, C: 60 ± 6.8	I: 28.3 ± 1.5, C: 29.0 ± 3.8	T2D	3	16	Aerobic: 40–65% VO_2max_, 30–60 min; Resistance: 2–4 sets of 12–20 repetitions, 40% to 60% of 1-RM	No	Leptin: I: −0.1 ± 0.95, C: −0.1 ± 1.4
Balducci, 2010 [39]	42; I: 36.4%, C: 45%	I: 60.6 ± 9.3, C: 61.1 ± 7.1	I: 30.5 ± 0.9, C: 30.9 ± 1.1	T2D	2	52	Aerobic: 70–80% VO_2max_, 40 min; Resistance: 80% 1-RM, 40 min	No	Adiponectin: I: 5.54 ± 3.6, C: 0 ± 3.6; leptin: I: −7.29 ± 8.33, C: −2.08 ± 8.33
Jorge, 2011 [40]	24; I: 66.6%, C: 66.6%	I: 57.90 ± 9.82, C: 53.41 ± 9.82	I: 31.24 ± 3.88, C: 29.59 ± 4.90	T2D	3	12	Aerobic: 30 min, intensity set individually acc. to lactate threshold; Resistance: 30 min, circuit training with 7 exercises	No	Adiponectin: I: 0.6 ± 4.76, C: −1.32 ± 4.76
Loimaala, 2009 [41]	48; 0%	I: 53.6 ± 6.2, C: 54.0 ± 5.0	I: 29.3 ± 3.7; 29.8 ± 3.6	T2D	4	104	Aerobic: 70–80% VO_2max,_ at least 30 min; Resistance: 3–4 sets with 10–12 repetitions, 80% 1-RM, at least 30 min	No	Leptin: I: −0.7 ± 3.9, C: 0.5 ± 3.6
Mendham, 2013 [42]	21; 0%	I: 39.5 ± 10.6, C: 36.1 ± 16.1	I: 31.6 ± 3.1, C: 34.5 ± 6.6	T2D	3	12	45 min sessions; Aerobic: 80–85% VO_2max_; Resistance: free-weights training with 3 exercises; additional boxing session	No	Adiponectin: I: −1.1 ± 3.71, C: −0.2 ± 2.96 leptin: I: −5.71 ± 7.7, C: 0.49 ± 15.06
Okada, 2010 [43]	38; I: 52.3%, C: 35.3%	I: 61.9 ± 8.6, C: 64.5 ± 5.9	I: 25.7 ± 3.2, C: 24.5 ± 2.9	T2D	3–5	12	Aerobic: 40 min, Resistance: 20 min	No	Adiponectin: I: 1.9 ± 3.89, C: 1.8 ± 3.25 leptin: I: 1.2 ± 3.3, C: 1.4 ± 4.9
**Aerobic Exercise**									
Aas, 2005 [44]	18; I: 33.3%, C: 33.3%	I: 59 (45–67), C: 53 (39–66)^a^	I: 29.8 (26.7–34.5), C: 30.0 (25.8–31.0)^a^	T2D	2	52	60 min, moderate intensity	Hypocaloric diet aimed at weight loss and improved metabolic control	Adiponectin: I: −1.4 ± 0.95, C: −1.1 ± 2.97 leptin: I: −1.17 ± 1.16, C: 3.17 ± 1.44
Balducci, 2010 [39]	40; 40%	I: 64.3 ± 8.1, C: 61.1 ± 7.1	I: 29.4 ± 1.1, C: 30.9 ± 1.1	T2D	2	52	70–80% VO_2max_, 60 min	No	Adiponectin: I: 5.97 ± 3.6, C: 0 ± 3.62 leptin: I: −4.17 ± 8.33, C: −2.08 ± 8.33
Boudou, 2003 [45]	16; 0%	I: 42.90 ± 5.20, C: 47.9 ± 8.35	I: 28.3 ± 3.90, C: 27.6 ± 4.30	T2D	3	8	2 sessions with continuous intensity: 75% VO_2max_ (45 min), 1 session with alternating intensity: 50–85% VO_2max_ (20 min)	No	Adiponectin: I: −0.3 ± 3.19, C: −0.25 ± 2.36, leptin: I: −0.45 ± 4.45; C: 0.14 ± 3.9
Dede, 2015 [46]	60; I: 50%; C: 53.3%	I: 52.5 ± 7.5, C: 55.5 ± 8.4	I: 30.8 ± 4.6, C: 30.2 ± 4.5	T2D	3	12	Sessions progressed from 15–20 min at 60% of VO_2max_ to 45 min at 75% of VO_2max_	No	Adiponectin: I: −0.6 ± 0.8, C: −0.2 ± 1.1 leptin: I: −2.8 ± 19.79, C: 1.3 ± 17.16
Giannopoulou, 2005 [47]	22; 100%	55.5 ± 1.7	35.9 ± 1.9	T2D	3–4	14	65–70% VO_2max_, 60 min	No	Adiponectin: I: 1.8 ± 1.37, C: 1.2 ± 1.95 leptin: I: −15.6 ± 8.77, C: −9.8 ± 8.77
Iishi, 2001 [48]	50; I: 60.9%; C: 59.3%	I: 56.0 ± 4.6, C: 57.9 ± 7.6	I: 26.2 ± 3.5, C: 25.4 ± 3.2	T2D	5	6	50% VO_2max_, 60 min	25- to 27-kcal/kg/d (54% to 58% carbohydrate, 22% to 24% protein, 18% to 20% fat)	Leptin: I: −2.6 ± 0.77, C: −1.1 ± 0.62
Jorge, 2011 [40]	24; I: 58.3%, C: 66.6%	I: 52.09 ± 8.71, C: 53.41 ± 9.82	I: 29.30 ± 2.09, C: 29.59 ± 4.90	T2D	3	12	Intensity set individually acc. to lactate threshold, 60 min	No	Adiponectin: I: 2.2 ± 5.0, C: −1.32 ± 4.76
Kadoglou, 2007a [49]	60; I: 56.6%, C: 60%	I: 59.33 ± 4.76, C: 63.82 ± 7.03	I: 32.1 ± 3.19, C: 31.99 ± 3.41	T2D	4	26	50–75% VO_2max_, 30–45 min	No	Adiponectin: I: −0.24 ± 0.95, C: 0.35 ± 0–93
Kadoglou, 2007b [50]	46; I: 65.21%, C: 60.86%	I: 56.91 ± 7.09, C: 60.32 ± 9.28	I: 31.14 ± 3.58, C: 28.96 ± 1.03	T2D	4	32	50–80% VO_2max_, 45–60 min	No	Adiponectin: I: 0.33 ± 1.15, C: −0.6 ± 0.8
Ku, 2010 [51]	31; 100%	I: 55.7 ± 7.0, C: 57.8 ± 8.1	I: 27.1 ± 2.4, C: 27.4 ± 2.8	T2D	5	12	3.6–5.2 METs (1 MET = 3.5 mL O_2_/kg/min), 60 min	No	Adiponectin: I: 2.9 ± 2.32, C: 1.99 ± 2.06 leptin: I: −3.73 ± 3.1, C: −0.1 ± 3.52
Marcell, 2005 [52]	34; 60.7%	I: 44.4 ± 6.5, C: 44.1 ± 9.5	I: 32.5 ± 5.3, C: 35.3 ± 3.7	IR	5	16	80–90% of age-predicted maximum heart rate (220 − age), 30 min	No	Adiponectin: I: 0.9 ± 1.0, C: −1.9 ± 1.1
Rokling-Andersen, 2007 [53]	85; 0%	45.1 ± 2.51	I: 28.5 ± 3.3, C: 28.5 ± 3.3	IFG, IGT	3	52	60–80% of measured maximum heart rate, 60 min	No	Adiponectin: I: −0.5 ± 7.35, C: −4.1 ± 8.82 leptin: I: −0.5 ± 4.7, C: 1.0 ± 7.61
Thompson, 2014 [54]	494; I: 66.0%, C: 64.0%	I: 60 ± 10, C: 60 ± 10	I: 31.6 ± 5.6, C: 31.5 ± 5.7	T2D	5	52	Low-intensity walking, 30 min	Hypocaloric diet aiming to produce 5–10% weight loss	Adiponectin: I: 0.96 ± 0.24, C: 0.5 ± 0.04
Zhang, 2017 [55]	32; I: 58,8%, C: 46,6%	I: 47.2 ± 10.5, C: 46.9 ± 11.1	I: 27.6 ± 3.2, C: 27.9 ± 3.8	T2D	5	12	70% of age-predicted maximum heart rate (220 − age), 60 min	No	Adiponectin: I: 1.2 ± 1.81, C: 0.4 ± 1.95 leptin: I: −3.3 ± 6.57, C: −2.1 ± 7.7
**Resistance Exercise**									
Brooks, 2007 [56]	62; I: 32.2%, C: 38.7%	I: 66 ± 2, C: 66 ± 1	I: 30.9 ± 1.1, C: 31.2 ± 1.0	T2D	3	16	60–80% of 1-RM, 3 sets with 8 repetitions, 35 min	No	Adiponectin: I: 2.3 ± 2.09, C: 1.99 ± 2.06
Jorge, 2011 [40]	24; I: 58.3%, C: C: 66.6%	I: 54.10 ± 8.94, C: 53.41 ± 9.82	I: 31.29 ± 4.08, C: C: 29.59 ± 4.90	T2D	3	12	60 min, circuit training with 7 exercises	No	Adiponectin: I: 0.68 ± 4.21, C: −1.32 ± 4.76
Ku, 2010 [51]	29; 100%	I: 55.7 ± 6.2, C: 57.8 ± 8.1	I: 27.1 ± 2.3, C: 27.4 ± 2.8	T2D	5	12	Elastic band exercises with 40–50% of maximal exercise capacity, 3 sets with 15–20 repetitions	No	Adiponectin: I: 2.3 ± 2.09, C: 1.99 ± 2.06 leptin: I: −1.02 ± 2.89, C: −0.1 ± 3.52
Lovrencic, 2015 [57]	140; I: 56.1%; C: 54.1%	I: 58.5 ± 4.8, C: 57.7 ± 6.2	I: 29.44 (4.67), C: 30.64 (4.54)	T2D	6	52	Strength exercises with light to medium intensity, 90 min	No	Adiponectin: I: −0.1 ± 6.42, C: −0.4 ± 3.31
**Exercise Advice**									
Balducci, 2009 [39]	40; 45%	I: 62.5 ± 7.1, C: 61.1 ± 7.1	I: 30.0 ± 1.0, C: 30.9 ± 1.1	T2D	NA	52	Counseling to perform low-level aerobic physical activity regularly	No	Adiponectin: I: −0.43 ± 6.63, C: 0 ± 3.6 leptin: I: −1.04 ± 2.89, C: −0.1 ± 3.52
Belalcazar, 2015 [58]	1397; I: 57%, C: 57%	I: 57.1 ± 7.1, C: 57.3 ± 7.3	I: 36.4 ± 6.4, C: 36.0 ± 5.9	T2D	NA	52	Advice to increase moderate intensity physical activity to at least 175 min per week	Hypocaloric diet aiming at 7% weight loss, 1200–1500 kcal/day if body weight <114 kg, 1500–1800 kcal/day if body weight ≥114 kg	Adiponectin: I: 0.5 ± 0.53, C: 0 ± 0.43
Corpeleijn, 2007 [59]	103; 45.5%	I: 55.6 ± 0.9, C: 57.8 ± 1.0	I: 29.8 ± 0.5, C: 29.3 ± 0.4	IGT	NA	52	Advice to perform 30 min of moderate intensity physical activity at least 5 times per week	Hypocaloric diet aiming at 5–7% weight loss, carbohydrate intake of at least 55% of total energy intake; total fat of 30 to 35% of total energy intake, with <10% energy intake of saturated fatty acids and cholesterol intake of <33 mg/MJ; protein 10 to 15% of total energy; dietary fiber at least 3 g/MJ	Adiponectin: I: −0.06 ± 0.63, C: −0.03 ± 0.55 leptin: I: −2.37 ± 1.3, C: 0.06 ± 0.93

^a^ Values expressed as median (95% confidence interval (CI)), ^†^ Units: µg/mL for adiponectin, ng/mL for leptin; T2D—type 2 diabetes, IFG—impaired fasting glucose, IGT—impaired glucose tolerance, VO_2max_—maximal oxygen consumption, MET—metabolic equivalent, kcal—kilocalories, MJ—mega-joule, BMI—body mass index, I: intervention group, C: control group, 1-RM: 1 repetition maximum.

**Table 2:** Results of subgroup analysis.

Adiponectin			Leptin	
	MD (95% CI), *p*-Value, I^2^-Value	Test for Subgroup Differences	MD (95% CI), *p*-Value, I^2^-Value	Test for Subgroup Differences
Intervention duration				
≤12 weeks	0.12 (0.02, 0.29), *p* = 0.57, I^2^ = 0%	*p* = 0.0009, I^2^ = 85.2%	−1.50 (−1.88, −1.12), *p* < 0.00001, I^2^ = 0%	*p* = 0.05, I^2^ = 74.7%
>12 weeks	0.49 (0.29, 0.68), *p* < 0.00001, I^2^ = 88%		−2.69 (−3.79, −1.58), *p* < 0.00001, I^2^ = 46%	
Dietary co-intervention				
Yes	0.40 (0.28, 0.51), *p* < 0.00001, I^2^ = 85%	*p* = 0.11, I^2^ = 60.2%	−2.60 (−3.72, −1.47), *p* < 0.00001, I^2^ = 92%	*p* = 0.01, I^2^ = 83.6%
No	0.99 (0.27, 1.71), *p* = 0.007, I^2^ = 82%		−0.87 (−1.65, −0.09), *p* = 0.03, I^2^ = 0%	
Training sessions/week				
≤3 times/week	1.70 (0.29, 3.12), *p* = 0.02, I^2^ = 76%	*p* = 0.24, I^2^ = 28.1%	−2.27 (−4.49, −0.05), *p* = 0.05, I^2^ = 75%	*p* = 0.68, I^2^ = 0%

## Supplementary Materials

The following are available online at http://www.mdpi.com/2076-3271/6/4/97/s1, Figure S1: Flow diagram of study selection, Figure S2: Risk of bias assessment. Search strategy for PubMed: (“training” OR “exercise” OR “physical activity”) and (“diabetes” OR “prediabetes” OR “insulin resistance” OR “impaired glucose tolerance” OR “impaired fasting glucose” OR “leptin” OR “adiponectin” OR “adipokines”) and (“randomized controlled trial” OR “randomized” OR “clinical trials as topic” OR “placebo” OR “randomly” OR “trial”) not (“animals” NOT “humans”).
