# Supplementary material for: Exercise Increases Adiponectin and Reduces Leptin Levels in Prediabetic and Diabetic Individuals: Systematic Review and Meta-Analysis of Randomized Controlled Trials
Source: Med Sci (Basel). 2018 Oct 30;6(4):97. doi: 10.3390/medsci6040097 (PMC6318757; doi:10.3390/medsci6040097)
Supplement: Supplementary file 1 [file medsci-06-00097-s001.pdf]

medical sciences

MDPI

Supplementary materials

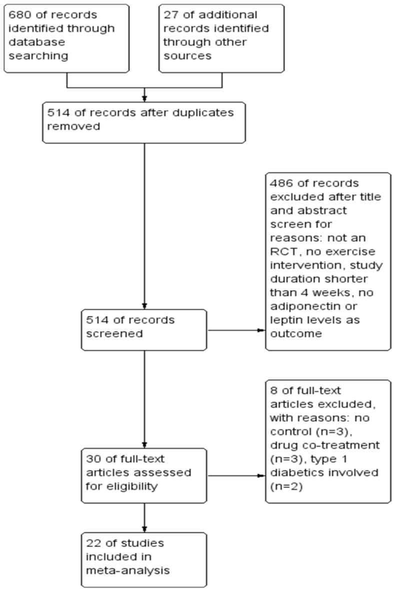

Figure S1. Flow diagram of study selection. RCT—randomized controlled trial.

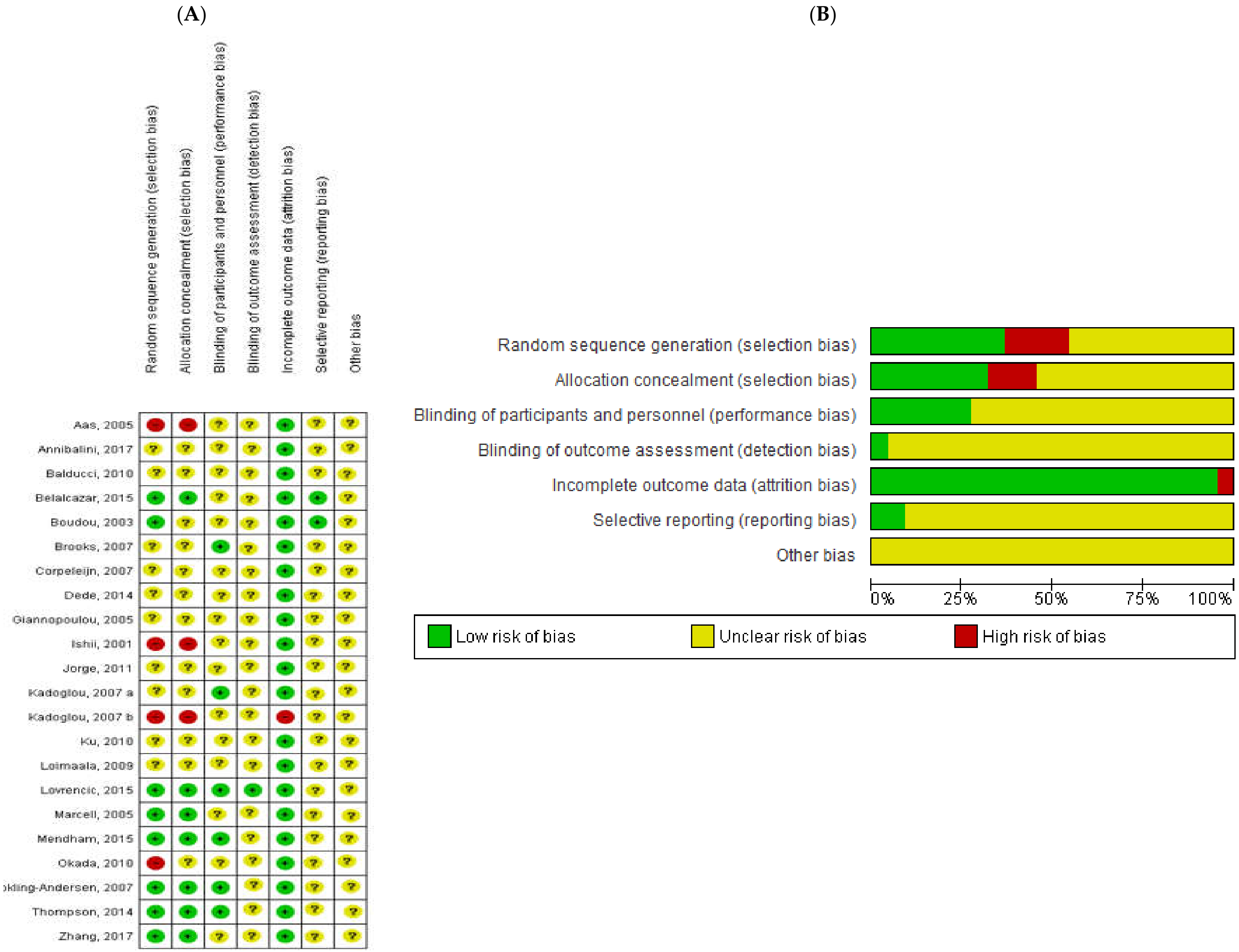
(A)
(B)
Random sequence generation (selection bias)
Allocation concealment (selection bias)
Blinding of participants and personnel (performance bias)
Blinding of outcome assessment (detection bias)
Incomplete outcome data (attrition bias)
Selective reporting (reporting bias)
Other bias
Aas, 2005
Annibalini, 2017
Balducci, 2010
Belalcazar, 2015
Boudou, 2003
Brooks, 2007
Corpeleijn, 2007
Dede, 2014
Giannopoulou, 2005
Ishii, 2001
Jorge, 2011
Kadoglou, 2007 a
Kadoglou, 2007 b
Ku, 2010
Loimaala, 2009
Lovrencic, 2015
Marcell, 2005
Mendham, 2015
Okada, 2010
kling-Andersen, 2007
Thompson, 2014
Zhang, 2017
0%
25%
50%
75%
100%
Low risk of bias
Unclear risk of bias
High risk of bias

Figure S2. Risk of bias assessment. Each bias domain was assessed for every study included in the meta-analysis and decided it reflected a low risk of bias (green), high risk of bias (red), or if insufficient information was provided so that the risk of bias was unclear (yellow). (A) Risk of bias in individual studies. (B) Summary of risk of bias.

© 2018 by the authors. Submitted for possible open access publication under the terms and conditions of the Creative Commons Attribution (CC BY) license (http://creativecommons.org/licenses/by/4.0/).
